# Intravesical migration of female urethral dilator: a case report of a new urologic emergency in the era of e-commerce

**DOI:** 10.1186/s12894-018-0398-4

**Published:** 2018-10-03

**Authors:** Andrea Mogorovich, Cesare Selli, Alessio Tognarelli, Francesca Manassero, Maurizio De Maria

**Affiliations:** Urology Unit, Department of Translational Research, University of Pisa, Via Paradisa 2, 56124 Pisa, Italy

**Keywords:** Bladder, Urethral dilator, Foreign bodies, Foreign body migration

## Abstract

**Background:**

The introduction of foreign bodies in the female urethra for auto-erotic stimulation or in case of psychiatric disorders is not uncommon. The occurrence of intravesical migration of these objects makes it necessary to remove it shortly after insertion, since after long term permanence complications are likely to occurr.

**Case presentation:**

A 47-year-old white female was referred at our Urology department for migration inside the bladder of a metallic urethral dilator used for sexual stimulation. An ultrasound study and an X-ray plate of the pelvis clearly visualized the presence of an object shaped like a rifle bullet located in the bladder. Twenty-four hours later, the patient reported its spontaneous emission through the urethra during micturition. This was confirmed by US and X-ray imaging.

**Conclusions:**

The retrieval of foreign objects introduced through body orifices with purpose of sexual gratification is a known urological expertise. Curiously, in the case reported, the patient was able to manipulate the object thus facilitating its correct orientation and passage outside the bladder during micturition. To the best of our knowledge this is the first case of documented spontaneous emission through the urethra of a sizable intravesical foreign body. Sexual gratification in females though the insertion of urethral dilators is a growing practice, as demonstrated by the broad proposal of such instruments on the web. Therefore, the occurrence of accidental intravesical displacement of such kind of foreign body is increasingly likely, and the Urologists must be aware of this possibility.

## Background

Introduction of foreign bodies in the female urethra is not uncommon, and the main reasons are auto-erotic stimulation, hygiene or psychiatric diseases. These objects may migrate inside the bladder due to the shortness of female urethra, its straight alignment and the fact that urethral meatus is usually not visible [1].

Usually, intravesical foreign bodies can be removed endoscopically shortly after their insertion, and they mostly consist in rigid objects such as pencils, ballpoint pens, pen casings, AAA batteries, paper clips with endless varieties [2].

Long term permanence leads to complications such as chronic urinary tract infection, bladder ulceration and formation of large size calculi, which can be found in patients with psychiatric disorders [3].

Emergent surgical management for injuries associated with eroticism, including the removal of foreign bodies, is increasing but still relatively uncommon, and there is a higher prevalence in men [4].

We report herein the case of a female patient who was referred at night from the Emergency Room for urologic consultation for intravesical migration of a conic-shaped urethral dilator bought on the Internet for self-gratification. The following day, before planned endoscopic extraction, she was able to self-manipulate retrogradely the dilator through the urethra outside the bladder. To our knowledge this is the first occurrence of such an event.

## Case presentation

**Fig. 1:**
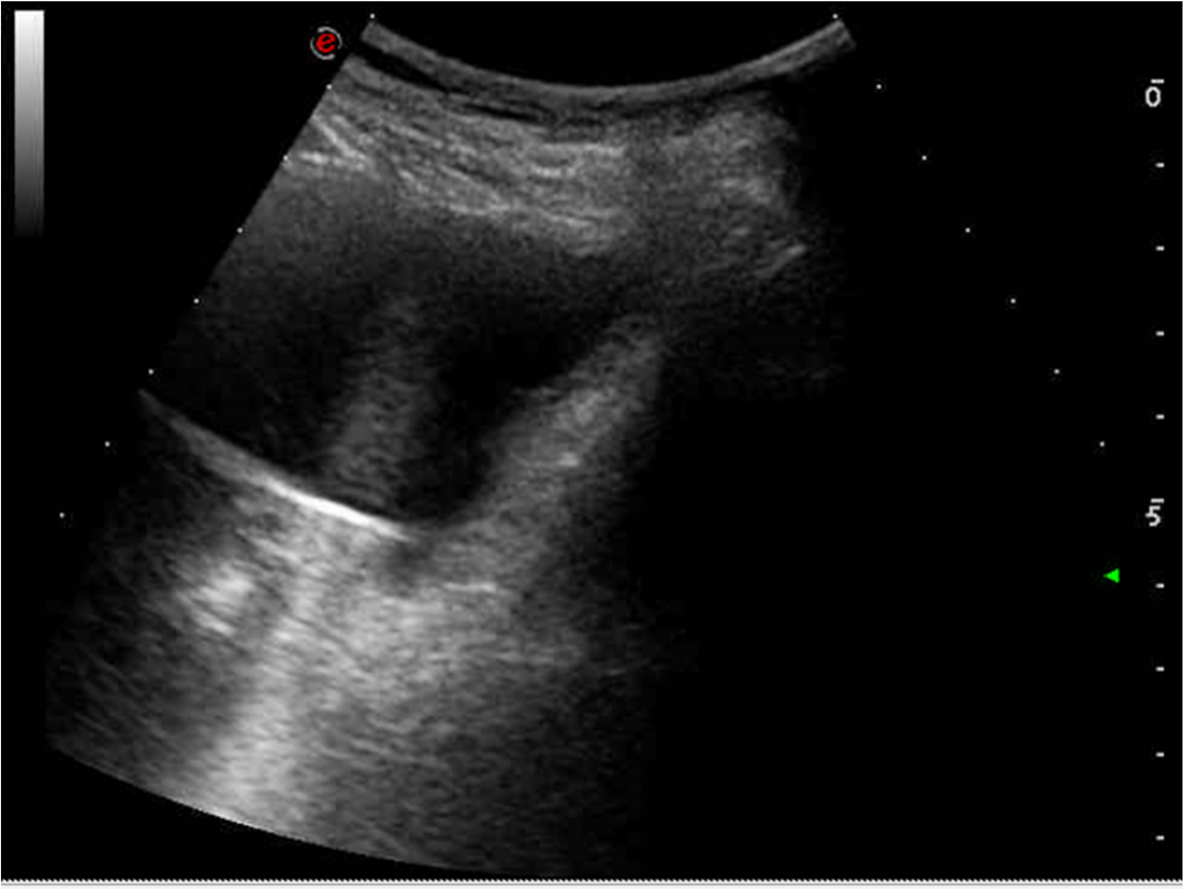
US scan revealing hyperechoic linear structure within the bladder

**Fig. 2:**
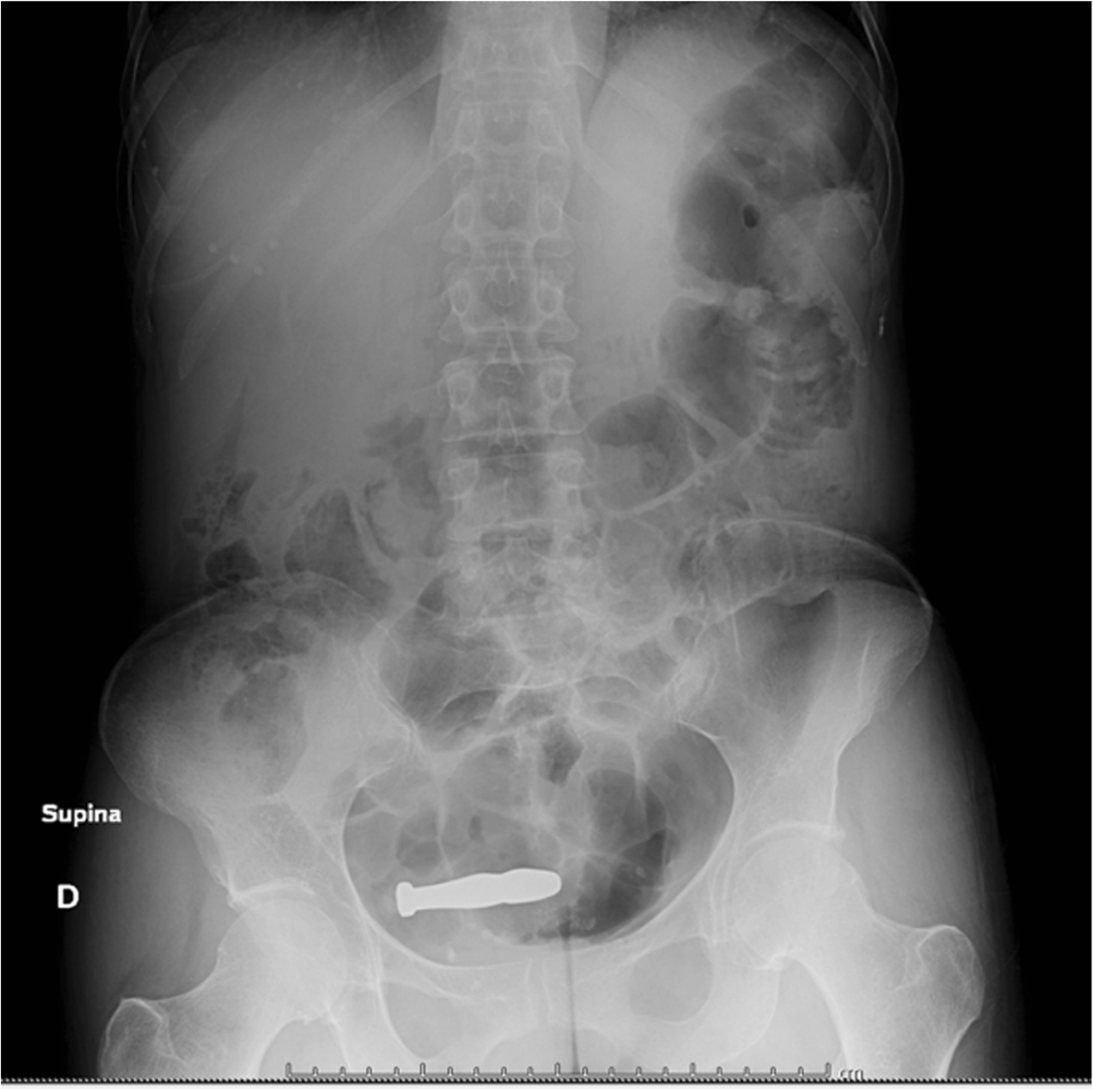
X-ray plan clearly shows the presence of an intensely radiopaque bullet-shaped object above the pubic symphysis

A 47-year-old white female was referred at 1 AM to our Urology department from the Emergency Room for admitted migration inside the bladder of a metallic urethral dilator used for sexual stimulation. The patient stated that she had bought the object through a dedicated internet site. An ultrasound study revealed a partially full bladder with an echogenic internal structure (Fig. 1). An X-ray plate of the pelvis clearly visualized the presence of a high-density object shaped like a rifle bullet about 6 cm long, placed obliquely above the pubic symphysis. It was referred by the Radiologist as “likely intrauterine device” (Fig. 2).

Since the patient had no symptoms, she opted to return home under oral antibacterial treatment with Ciprofloxacin, and endoscopic extraction was planned in 2 days time, with the program of introducing through the urethra under sedation a 24 F nephroscope and to extract the dilator placing it in line with the instrument axis and retrieving it with a 3-pronged rigid grasper.

**Fig. 3:**
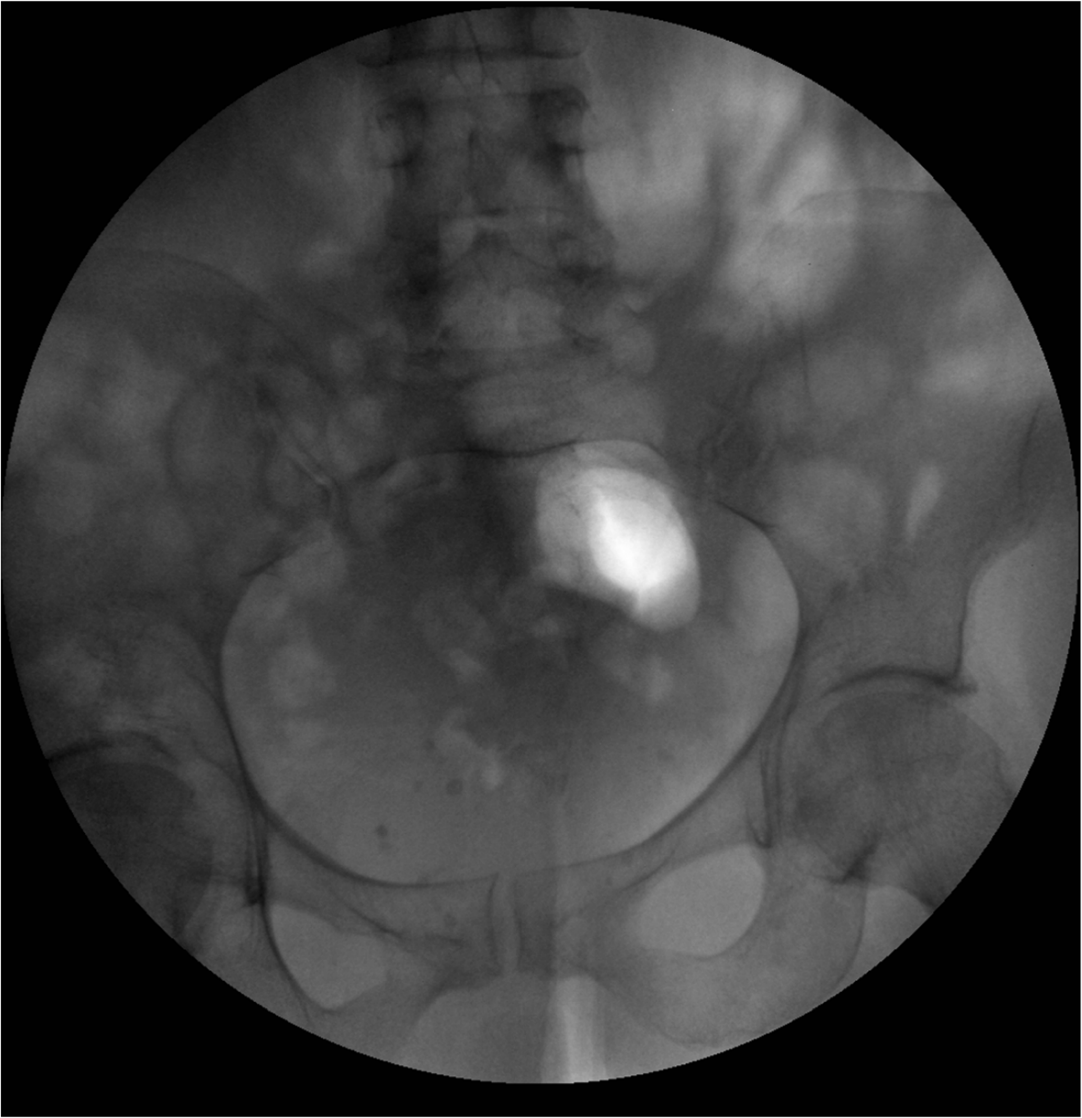
X-ray of the pelvis after foreign body exit

The following day, when contacted by telephone again, the patient refused hospitalization, stating that she had be able to self-manipulate retrogradely the dilator through the urethra outside the bladder (Table 1). An US and X-ray study of the pelvis confirmed the absence of the foreign body (Fig. 3).

**Table 1:** Summary of the clinical case

When	Patient details	Patient’s concern	Management	Interventions
Initial diagnostic assessment during the night	47-yo white female reffered to our Department	Migration inside the bladder of a metallic urethral dilator during sexual stimulation	US and X-Ray	Oral antibacterial treatment, discharged...planned endoscopic removal after 2 days
After 1 day	–	The patient said was able to self-manipulate retrogradely the dilator through the urethra outside the bladder	US and X-Ray	Discharged home

## Discussion and conclusions

The retrieval of foreign objects introduced through body orifices with purpose of sexual gratification is a known urological expertise, and this practice is defined as polyembolokoilamania [5]. However the present case presents two points of interest.

**Fig. 4:**
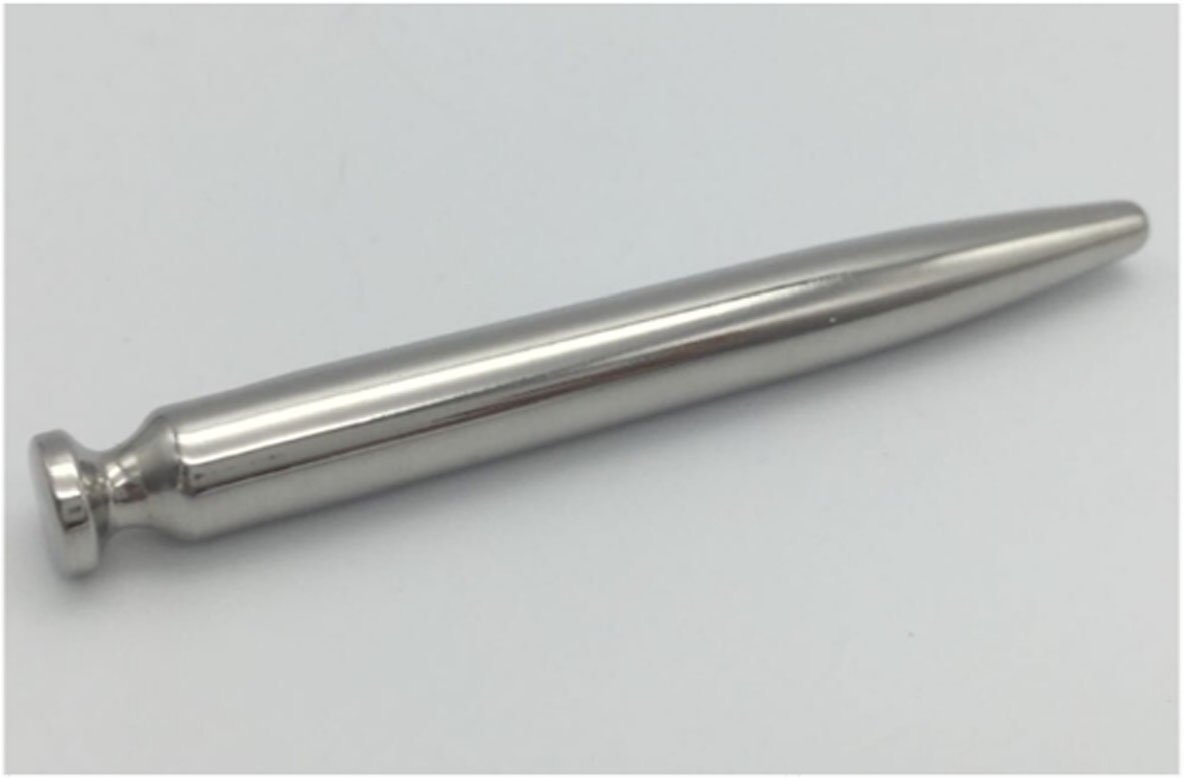
Example of an urethral dilator available for purchase on e-commerce similar in shape and size to that one used in the present case report

The first one is that sexual gratification in females though the insertion inside the urethra of elongated smooth objects of tapered shape is a practice more common than previously believed, particularly in some cultures of the far East. It is not a coincidence that the largest published series comes from such a geographical area [2] and that Asian e-commerce sites under the heading “urethral dilators” offer such devices (Fig. 4). Interestingly some present at the larger end attached a metallic ring, evidently both for easier use and to avoid the possibility of upwards migration of the metallic object. Therefore it is likely that similar cases have occurred, but to the best of our knowledge have not been reported in the medical literature.

The second rather unique characteristic of the present case report is that the patient has been able to self-manipulate the object outside the bladder though the urethra, showing high manual dexterity. Repeated urethral dilatations evidently facilitated this uncommon maneuver, and we are unaware of such an occurrence.

Metallic bullet-shaped urethral dilators are presently available on e-commerce for sexual gratification by transurethral insertion. This increased availability makes the occurrence of accidental intravesical displacement more likely, and the Urologists must be aware of this possibility. Some of these objects have a “safety” metallic ring connected to the wider end, but should it be absent endoscopic retrieval must be performed.

Paradoxically the object weight and shape in the present case allowed its quite extraordinary extraction by the patient herself.
